# Targeting of the Pilosebaceous Follicle by Liquid Crystal Nanocarriers: In Vitro and In Vivo Effects of the Entrapped Minoxidil

**DOI:** 10.3390/pharmaceutics12111127

**Published:** 2020-11-22

**Authors:** Massimo Fresta, Antonia Mancuso, Maria Chiara Cristiano, Konrad Urbanek, Felisa Cilurzo, Donato Cosco, Michelangelo Iannone, Donatella Paolino

**Affiliations:** 1 Department of Health Sciences, University “Magna Græcia” of Catanzaro, Campus Universitario “S. Venuta”, viale Europa, 88100 Germaneto (CZ), Italy; fresta@unicz.it (M.F.); antonia.mancuso@unicz.it (A.M.); donatocosco@unicz.it (D.C.); 2 Department of Experimental and Clinical Medicine, University “Magna Græcia” of Catanzaro, Campus Universitario “S. Venuta”, viale Europa, 88100 Germaneto (CZ), Italy; mchiara.cristiano@unicz.it (M.C.C.); urbanek@unicz.it (K.U.); 3 Department of Pharmacy, University of Chieti—Pescara “G. d’Annunzio”, Via dei Vestini 31, 66100 Chieti, Italy; felisa.cilurzo@unich.it; 4 National Council of Research (CNR), The Institute for Biomedical Research and Innovation, viale Europa, 88100 Germaneto (CZ), Italy; michelangelo.iannone@cnr.it

**Keywords:** liquid crystal nanocarriers, topical drug delivery, in vivo, in vitro, transfollicular, minoxidil, pilosebaceous follicle, alopecia

## Abstract

The topical administration of active compounds represents an advantageous strategy to reach the various skin components as well as its appendages. Pilosebaceous follicles are skin appendages originating in the deeper skin layers. They are very difficult to target, and hence higher active dosages are generally required to achieve effective biological responses, thus favoring the rise of side effects. The aim of this work was to design a supramolecular colloidal carrier, i.e., a liquid crystal nanocarrier, for the selective delivery of active compounds into the pilosebaceous follicle. This nanocarrier showed mean sizes of ~80 nm, a good stability, a negative surface charge, and great safety properties. In vitro studies highlighted its ability to contain and release different substances and to successfully permeate the skin. Minoxidil was encapsulated in the nanocarriers and the in vivo biological effect was compared with a conventional dosage form. Minoxidil-loaded liquid crystal nanocarrier was able to selectively reach the pilosebaceous follicle, thus allowing an increased biological effectiveness of the delivered active in terms of biological response, duration of the biological effects, and reduction of collaterals. Our investigation showed that liquid crystal nanocarriers represent a promising device for the treatment of different pilosebaceous follicular impairments/diseases.

## 1. Introduction

In recent years, the skin has been considered one of the most advantageous sites for the administration of active compounds. However, the penetration into the deeper skin layers is severely limited due to the barrier function of the stratum corneum [1]. For this reason, both in pharmaceutical and cosmetic fields, a number of active compounds have a reduced biological action due to their inability to cross the stratum corneum and hence to reach the target site.

The pilosebaceous unit (comprising hair follicle, hair shaft, arrector pili muscle, and sebaceous gland) originates in the dermis and represents a versatile penetration route beside the stratum corneum [2]. Because the sebaceous glands are more permeable than corneocytes, active compounds can also reach the deeper skin layers passing through them or crossing the epithelium of the follicular sheath [3]. Recent studies highlighted the potential of this transappendageal pathway in transdermal drug delivery [4]. However, following a topical administration, only a small fraction of the formulation reaches the hair follicles that occupy merely 0.1% of the total surface area of the skin. Therefore, greater dosages are usually required to obtain therapeutic concentrations, thus prompting the rise of side effects [5]. Furthermore, various diseases, i.e., mycoses [6], acne vulgaris [7], folliculitis [8], seborrheic dermatitis [9] involve mostly/directly the pilosebaceous follicles, requiring a precise delivery of active compounds at the level of pilosebaceous follicles rather than the unspecific spreading on the large skin surface.

For these reasons various topical drug delivery systems (TDDSs) were investigated to improve the concentration of active compounds into the skin and specifically at the level of the pilosebaceous follicles, thus allowing a reduction of systemic side effects [10,11,12,13]. In particular, niosomes loaded with minoxidil showed no permeation through the skin of hairless mice, demonstrating a greater suitability for topical skin application in comparison with conventional topical formulation [14]. Considering the similarity between the lipids of the investigated formulations and those of sebum, which are abundantly present in the follicle, different lipid nanoparticle dispersions, such as solid lipid nanoparticles or nanolipid carriers, have also been proposed as a valid strategy for follicular targeting. Unfortunately, recent studies have demonstrated the lack of a significant superiority of these nanocarriers compared to other topical formulations, due to their unspecific follicular penetration and the contemporaneous loss of the entrapped active ingredients on the skin surface [3]. Further details on their stability following topical application are still necessary to clarify, due to the possible alteration of the structure that can occur during the penetration through the upper layers of the skin, no longer allowing the desired site-specific delivery [3]. Another aspect that has to be taken into consideration for the evaluation of solid lipid nanoparticles is the absence of alcohol in their formulation, which, on one hand, can improve the cutaneous tolerance thus avoiding skin irritation phenomena, but on the other, can limit the follicular delivery of active compounds.

Another nanocarrier investigated for achieving a selective delivery towards pilosebaceous follicles was the liposome. In particular, liposomes bearing positive charges on their surface were proposed [15]. Unfortunately, as subsequently reported [16], positively charged liposomes evidenced a greater localization at the level of the pilosebaceous unit, but also a poor retention of the entrapped active compound during their passage through the follicles. It is noteworthy that electrostatic interactions between carriers and the negatively charged skin surface can occur, thus increasing the accumulation of this nanocarrier in the upper skin layers while reducing the permeation of active compounds into the deeper layers [3].

Therefore, while the follicular targeting represents a desired strategy for topical skin delivery of active compounds, a challenge remains regarding new formulations to be developed to reach this goal. To meet this need, the present study investigates an innovative liquid crystal nanocarrier (LCN), able to self-assemble in a supramolecular structure, which can provide a site-specific delivery at the level of the pilosebaceous follicles using minoxidil as a model of active compound.

Minoxidil is a pyrimidine derivative, able to significantly influence the hair re-growth through different mechanism of action, following its topical administration. Minoxidil, a potassium channels opener, can stimulate the vascular endothelial growth factor and the synthesis of prostaglandin in the dermal papilla [17,18,19]. Minoxidil is poorly permeable through the skin and is available on the market as a conventional dosage form containing 5% *w*/*v* of active in a mixture of propylene glycol, ethanol, and water, to enhance the permeation. Unfortunately, during the topical administration, ethanol evaporates, thus generating insoluble minoxidil crystals with a subsequent reduction of its skin uptake. Moreover, the recommended twice-daily application of the traditional hydro-alcoholic solution generally causes serious side effects, i.e., skin irritation, rash, or dermatitis [14,19]. Rarely, the topically applied minoxidil can also be systematically absorbed, causing adverse effects in heart function, especially in young patients [20]. Based on these considerations, suitable and innovative topical formulations are needed to increase the follicular bioavailability of minoxidil, thus improving its biological efficacy and minimizing side effects.

Therefore, our investigation on LCN is focused on the increase of the follicular bioavailability of topically applied minoxidil in order to achieve greater therapeutic efficacy. In particular, physicochemical and technological studies have been performed. The toxicity of the LCN has been tested on a keratinocyte cell line and healthy volunteers to evaluate the LCN safety for topical application. In vitro and in vivo studies have been carried out to test the ability of carriers to effectively permeate the skin and reach the pilosebaceous unit. LCN was compared to other carriers known for their ability to deliver active substances into the skin. Finally, efficacy tests were performed on rats. The results showed that LCN can selectively reach the pilosebaceous follicle and thus significantly improve the therapeutic efficacy of minoxidil.

## 2. Materials and Methods

### 2.1. Materials

Phospholipon^®^ 90 G (PL^®^ 90G) was supplied by Lipoid GmbH (Ludwigshafen, Germany). NCTC2544 cells were provided by the Instituto Zooprofilattico of Modena and Reggio Emilia (Reggio Emilia, Italy). Dulbecco’s Modified Eagle Medium (DMEM), glutamine, Trypsin/ethylenediaminoaceticacid (EDTA) (1 ×) solution, penicillin (100 µL/mL)-streptomycin (100 µg/mL) solution (1% *v*/*v*), and fetal bovine serum (FBS) were purchased from GIBCO (Invitrogen Corporation, Giuliano Milanese, Milan, Italy). Glyceryl monooleate (Monomuls^®^ 90-O18) was obtained from Cognis S.p.A. (Fino Mornasco, Como, Italy). The following chemicals were purchased from Sigma-Aldrich (Milan, Italy): Minoxidil (≥99% TLC), oil red O and fluorescein sodium salt (used as markers), poloxamer 407 (pluronic^®^ F127), trilaurin, cremophor EL^®^, n-butanol, sodium cholate hydrate (SC), 1,2-dimyristoyl-sn-glycero-3-phospho-rac-(1-glycerol) sodium salt (DMPG), trypan blue dye solution (0.4% *v*/*v*), amphotericin B solution (250 µg/mL), phosphate buffer saline (PBS) solution, 3-[4,5-dimethylthiazol-2-yl]-3,5-diphenyltetrazolium bromide salt (used for MTT test), sodium dimethyl sulfoxide (DMSO). Span 80 was purchased by A.C.E.F. S.p.A. (Fiorenzuola D’Arda, Piacenza, Italy). Double distilled pyrogen-free water was used throughout the various experiments. All materials and solvents (Carlo Erba, Milan, Italy) used in this study were of analytical grade and did not require further purification.

### 2.2. Preparation of Monoglyceride-Based Liquid Crystal Nanocarrier (LCN)

The LCNs were made up of monoglycerides (Monomuls^®^ 90-O18), phospholipids and the non-ionic surfactant poloxamer 407 (BASF trade name-Pluronic^®^ F127). Briefly, Monomuls^®^ 90-O18 (90 mg) and the anionic phospholipid DMPG (1 mg) were placed in a Pyrex^®^ glass vial and dissolved in 5 mL of chloroform/ethanol mixture (10:90 *v*/*v*). Poloxamer 407 (50 mg) was dissolved in water (10 mL) in a second glass vial. The organic phase was added dropwise to the aqueous phase, under continuous stirring (10,000 rpm) using the Ultraturrax T25 homogenizer (IKA^®^—Werke GmbH & Co. KG, Staufen, Germany). Once the organic phase has been added, ten homogenization cycles (thirty-second each) at 15,000 rpm with 1 min rest between them were carried out. Finally, the formulation was maintained under continuous stirring (Orbital Shaker KS 130 Control, IKA-WERKE, Staufen, Germany) for 36 h at room temperature to remove any trace of the organic solvent. For the preparation of probe-loaded LCNs, 200 µL of the different probe solutions by presenting different physical-chemical features (i.e., red oil and fluorescein, lipophilic and hydrophilic, respectively) were added (0.1% *w*/*v*) to the appropriate phase according to their solubility. Minoxidil (0.5% *w*/*v*) was added to the organic phase to obtain the active compound-loaded LCNs.

### 2.3. Preparation of Transfersomes^®^

Transfersomes^®^ were prepared according to the thin layer evaporation method, as previously described [21]. PL^®^ 90G (88 mg) and SC (12 mg) were placed in a Pyrex^®^ glass vial and dissolved in absolute ethanol. The organic solvent was evaporated using Rotavapor^®^ R-210 (Büchi Italia, Milan, Italy) under a slow nitrogen flux and a thin lipid layer was obtained along the walls of the vial. The obtained film was stored overnight at 4 °C to remove any residual trace of solvent and it was subsequently hydrated with 6 mL of water/ethanol (93:7 *v*/*v*) mixture, under continuous stirring at 700 rpm for 15 min (Orbital Shaker KS 130 Control, IKA-WERKE, Staufen, Germany). The resulting vesicular suspension was extruded using polycarbonate filters (400 nm and 200 nm) by a Lipex Extruder^TM^ (Lipex biomembranes Inc., Vancouver, BC, Canada) to obtain unilamellar vesicles having mean sizes and polydispersity index suitable for topical skin delivery [22]. Transfersomes^®^ were finally put in a bath at 40 °C for 2 h to stabilize. For the preparation of probe-loaded transfersomes^®^, the hydrophilic probe (fluorescein) was solubilized in the aqueous phase used for the film hydration and the hydrophobic one (red oil) was solubilized in the organic phase. Minoxidil-loaded vesicles were prepared adding the active compound (0.5% *w*/*v*) in the organic phase.

### 2.4. Preparation of Solid Lipid Nanoparticles

The preparation of solid lipid nanoparticles (SLNs) was carried out following a microemulsive method. Briefly, trilaurin was put into a glass vial and melted at 70 °C. Subsequently, cremophor EL^®^, Span^®^ 80 and water were added to the preparation at room temperature. Following the dissolution of the various components, n-butanol was added drop by drop until a clear microemulsion was formed. Finally, 4 °C cooled water was added to the microemulsion thus obtaining SLNs. The residual organic solvent was evaporated off by slow magnetic stirring for 72 h at room temperature. In order to obtain probe-loaded SLNs, fluorescein and red oil were added to the aqueous phase or to the organic phase, respectively. Minoxidil-loaded SLNs were obtained adding the active compound (0.5% *w*/*v*) in the organic phase before the formation of microemulsions.

### 2.5. Physico-Chemical Characterization of Nanocarriers

Mean size and polydispersity index of nanocarrier samples were evaluated by Zetasizer Nano ZS (Malvern Instruments Ltd., Worcestershire, UK), a dynamic light scattering spectrophotometer, equipped with a laser diode with a rated output of 4.5 mV, operating at 670 nm, with a backscattering angle of 173°. The Zetasizer Nano ZS also incorporates a zeta potential analyzer that uses electrophoretic light scattering to evaluate any surface charges of the nanocarriers. Before the analysis, each sample was placed in polycarbonate cuvettes and suitably diluted with an isotonic solution to avoid multi-scattering phenomena. The various measurements were carried out in triplicate on three different batches (each one of 10 determination). Results were expressed as the mean of three measurements ± standard deviation [23].

Confocal micrographs provided information on the shape of the carrier. The analyses were performed using confocal laser scanning microscopy (Leica TCS SP2 MP, Buccinasco, Milan, Italy) operating at λ_exc_ = 496 nm and λ_em_ = 519 nm. A scanning resolution of 4096 × 4096 pixel was used with a laser beam Ar/Kr of 75 mW, equipped with fluorescein filters. Micrographs were recorded by a suitable software Leica using digital direct access control knobs for multi-dimensional acquisitions. The oil immersion lens 63× was used during the analysis.

### 2.6. Turbiscan Lab^®^ Expert Analysis

Formulations were submitted to Turbiscan Lab^®^ Expert (Formulaction, L’Union, France) analysis to evaluate their stability. Samples were placed into cylindrical glass tubes and measurements were carried out using a pulsing near infrared LED set at a wavelength of 880 nm. The light flux transmitted (T) and backscattered (BS) through the sample was detected in percentage relative to two standards (suspension of polystyrene latex and silicone oil) as a function of the sample height (mm). Any variations of backscattering and transmission signals were assessed for 3 h at 24 ± 1 °C [21].

### 2.7. Evaluation of Drug Loading Capacity

The entrapment efficiency of minoxidil in the various nanocarriers was evaluated using the ultracentrifugation method. The colloidal formulations were transferred into polycarbonate tubes and they were centrifuged (Avanti 30, Beckman, Fullerton, CA, USA) at 4 °C and 28,000 rpm. The supernatants were withdrawn and analyzed using high performance liquid chromatography (HPLC) apparatus. The entrapment efficiency was calculated as the percentage of the added active compound that becomes associated within the carrier, using the following equations:

(1) EE=[(Dt−Ds)/Dt]×100

(2) EE=[(De)/Dt]×100

where *Dt* is the total amount of drug added during the preparation of nanocarriers, *Ds* is the drug amount in the supernatant, and *De* is the amount of the encapsulated drug recovered in the pellet [24]. Data were acquired and processed with Galaxie^®^ chromatography manager software (Varian Inc., Palo Alto, CA, USA). The values of entrapment efficacy (EE) obtained from the two different equations differed by less than 3%.

### 2.8. HPLC Analysis

Minoxidil content was determined by reverse-phase chromatography using a HPLC system (Varian Inc., Palo Alto, CA, USA) equipped with a 200-2031 Metachem online degasser, a M210 binary pump, a G1316A thermostated column compartment, a ProStar 410 autosampler, and a 25 µL CSL20 Cheminert Sample Loop injector. The Galaxie^®^ chromatography manager software (Varian Inc., Palo Alto, CA, USA) was used to process data. The chromatographic separation was carried out at room temperature using a GraceSmart RP C18 column (4.6 × 250 mm, 5 μm particle size, Alltech Grom GmbH, Rottenburg-Hailfingen, Germany). The mobile phase used for the analysis was a mixture of methanol/water (80:20 *v*/*v*). The flow rate was 1.0 mL/min and UV detection was set at 231 nm.

### 2.9. Cell Culture

Cytotoxic effects of LCNs were evaluated on human keratinocytes (NCTC2544) with the trypan blue dye exclusion assay (cell mortality) and the MTT dye test (cell viability). NCTC2544 cells were incubated in plastic culture dishes (100 × 20 mm) in a Water-Jacketed CO_2_ incubator at 37 °C (5% CO_2_) using minimum essential medium (D-MEM) supplemented with glutamax, streptomycin (100 µg/mL)-penicillin (100 µL/mL) solution (1% *v*/*v*), amphotericin B (250 µg/mL), and FBS (10% *v*/*v*). The culture medium was replaced with fresh medium every 48 h. When ~80% of cellular confluence occurred, cells were washed with phosphate buffer solution (PBS) and were harvested by 2 mL of trypsin/EDTA (1×) solution to be transferred into centrifuge tubes containing cell culture medium. The tubes were centrifuged using Megafuge 1.0 (Heraeus Sepatech, Osterode/Harz, Germany) at 1200 rpm for 10 min at room temperature and the obtained pellet was re-suspended in the culture medium.

### 2.10. Evaluation of Cytotoxicity

The cells were seeded at a density of 3 × 10^4^ cells/mL into 12-well plastic culture dishes and at a density of 1.8 × 10^3^ cells/100 µL in 96-well plastic culture dishes to carry out the dye exclusion assay and the MTT dye test, respectively. After 24 h of incubation, the culture medium was replaced with fresh medium and LCNs were added at final scalar dilution (0.01, 0.1, 1.0, 10.0 µM) in lipid concentration and cells were incubated for 24 h, 48 h, and 72 h, thus evaluating both the dose-dependent and the time-dependent effect. For the trypan blue dye exclusion assay, untreated NCTC2544 cells were used as control. After the treatment, the cells were harvested using 2 mL of trypsin/EDTA (1×) solution and transferred into a plastic centrifuge tube containing 4 mL of culture medium. The tube was centrifuged with a Megafuge 1.0 (Heraeus Sepatech, Osterode/Harz, Germany) at 1200 rpm for 10 min at room temperature. The supernatant was withdrawn, and the pellet was re-suspended in 200 µL of trypan blue buffer for 30 s. The number of blue stained cells (dead cells) was determined with an hematocytometer chamber using an optical microscope (Labophot-2, Nikon, Japan). The following equation was used to evaluate the percentage of cell mortality:

(3) $$\%\;Cell\;death=\;\frac{Dc}{Tc}\times 100$$

where *Dc* is the number of dead cells (blue stained cells) and *Tc* is the total number of cells.

The obtained data were correlated to those of cellular viability obtained from MTT assay [24]. Following the treatment of keratinocyte cells with LCNs, each well was treated with 10 µL of MTT solution (5 mg/mL dissolved in PBS buffer) and keratinocytes were further incubated for 3 h to allow the formation of violet formazan crystals by living cells. The culture medium was successively removed, and the obtained formazan crystals were dissolved with 200 µL of dimethylsulfoxide/ethanol (1:1 *v*/*v*) mixture. Finally, the plates were gently shaken (KS 130 Control, IKA^®^ Werke GMBH & Co., Staufen, Germany) for 20 min at 230 rpm, before the analysis. Cell viability of samples was evaluated using an ELISA microplate reader (Labsystems mod. Multiskan MS, Midland, ON, Canada) that was set at λ_exc_ 570 nm and λ_em_ 670 nm for the emission. The following equation was used to evaluate the cell viability percentage, directly proportional to the amount of formazan crystals formed:

(4) $$\%\;Cell\;viability=\;\frac{At}{Au}\times 100$$

where *At* is the absorbance of treated cells and *Au* is the absorbance of the untreated ones.

Values of cell mortality and cell viability were the average of six different experiments ± standard deviation.

### 2.11. Release Rate from Nanocarriers

Franz-type diffusion cells were used to evaluate the release rate of fluorescein and red oil from the various nanocarriers, i.e., LCNs, transfersomes^®^, and SLNs. Cellulose acetate membranes (Spectra/Por, Spectrum Laboratories Inc., Eindhoven, The Netherlands), with a molecular cut-off of 10,000 Da, were used [21]. The receptor of Franz diffusion cells was filled with a water:ethanol (50:50 *v*/*v*) mixture as the release medium. Every hour, 200 µL were collected and replaced with fresh medium. The released amounts of the two probes were determined over 24 h by using an HPLC and the percentage of released drug was evaluated according to the following equation:

(5) $$\mathrm{Drug}\;\mathrm{release}\;\left(\%\right)=\frac{drug\;rel}{drug\;load}\times 100$$

where *drug rel* is the amount of released drug at a certain time and *drug load* is the quantity of entrapped drug within the nanocarriers.

### 2.12. In Vitro Percutaneous Permeation

#### 2.12.1. Isolation of SCE-Membranes

Franz-type diffusion cells were used to evaluate the percutaneous permeation of the various nanocarriers through human stratum corneum and viable epidermis membranes (SCE-membranes). The membrane sheets were isolated according to the procedure described by Kligman and Christophers (1963) [25], using three different batches of human skin obtained from abdominal reduction surgery of adult male subjects (mean age 34 ± 11 years). Briefly, the subcutaneous fat was surgically removed with a scalpel and the skin was immersed in distilled water at 60 ± 1 °C for 2 min. Stratum corneum and epidermis membranes were finally peeled off from the underlying dermis, thus obtaining SCE-membranes, which were dried in desiccator (25% relative humidity) and stored at 4 °C until use. The barrier integrity of SCE-membranes was checked through a pilot study, using tritiated water as permeating agent and evaluating its permeability coefficient (Kp) through membrane sheets. The resulting Kp = 1.7 ± 0.3 × 10^−3^ cm/h was in agreement with results previously reported for intact skin barriers [24]. The integrity of SCE membranes was confirmed before their use through transepithelial electrical resistance (TEER) evaluations [26].

#### 2.12.2. Percutaneous Permeation

In order to compare LCNs with other existing topical drug delivery systems (transfersomes^®^ and SLNs), in vitro studies were carried out by analyzing the percutaneous permeation features of the various probe-loaded nanocarriers.

Percutaneous permeation studies were carried out using SCE-membranes mounted horizontally between the donor and receptor compartment of Franz diffusion cells (Laboratory Glass Apparatus Inc., Berkeley, CA, USA), with the stratum corneum side up. Each donor was filled with 200 µL of the investigated nanocarriers loaded with one of the two probes (fluorescein or red oil) at 0.1% *w*/*v*. The experiments were carried out in non-occlusive conditions with Franz diffusion cells thermostated at 35 ± 1 °C by means of a circulating water bath. Samples of the receptor phase (1 mL) were collected every hour up to 24 h by using an FC 204 fraction collector connected to a Minipuls 3 peristaltic pump (Gilson Italia S.r.l., Milan, Italy). The system included automatic collection of samples. The withdrawn volume was automatically replaced at the scheduled times by the same amount of fresh receptor phase. The collected samples were analyzed by HPLC to evaluate the amount of probe permeated through SCE-membranes. Six different experiments were carried out for each formulation and the results were expressed as the mean values ± standard deviation.

### 2.13. Animals

Male Wistar rats (220 ± 10 g) were purchased from Harlan (Correzzana, Milan, Italy). Animals were housed in standard conditions of humidity and temperature (65% RH, 25 ± 1 °C) in metabolic cages and maintained at dark/light cycle of 12 h with water and food ad libitum. The experiments were carried out in accordance with all the principles and procedures outlined by the local Ethical Committee and the European Communities Council Directive of 24 November 1986 (86/609/EEC).

### 2.14. In Vivo Skin Distribution of Nanocarriers

These experiments were aimed to the evaluation of the skin distribution of LCNs in comparison with the other two investigated nanocarriers (SLNs and transfersomes^®^), well-known for their topical use. Before starting the experiment, rats were shaved by electric shaver and they were distributed in three groups (*n* = 9, three for each scheduled pick-up time). Fluorescein-labeled nanocarriers (200 µL, 0.2% *w*/*v*) were applied on the back of rats. A gelled probe solution was also applied to the rat skin as a control. Each preparation was administered in duplicate. After 1 h, 12 h, and 24 h from the application of the various nanocarrier formulations, skin samples were excised and stored in a buffered formalin to investigate the path of fluorescein through the whole skin. Skin sections were finally dissected by microtome (VT1000S Leica Microsystems Nussloch GmbH, Germany) in 50 µm slices and they were fixed on glass slides. Micrographs were obtained by using the confocal laser scanning microscopy (CLSM) [27]. The instrument operated at λ_exc_ = 496 nm and λ_em_ = 519 nm for the fluorescein probe. The different confocal images were obtained by the software Leica using digital direct access control knobs for multi-dimensional acquisition.

### 2.15. In Vivo Skin Distribution of Minoxidil

Before the anti-hair loss/hair re-growth feature evaluation, the various minoxidil-loaded nanocarriers were tested on rats (*n* = 9) to evaluate the active compound skin distribution in comparison with a hydro-alcoholic formulation of the active compound. The active compound distribution was monitored by CLSM thanks to the fluorescent emission of minoxidil. A sample (200 µL) of the various minoxidil-loaded nanocarrier formulations (0.5% *w*/*v*) was applied on the skin of rats. After 2 h, 6 h, and 24 h from application, samples of the skin application sites were excised, treated as previously reported (see Section 2.14) and observed using CLSM.

The distribution of the minoxidil amount in the different skin layers was also evaluated by HPLC. The study was carried out on 3 animals for each group. Following the application for 24 h of the various formulations (200 µL), the sites of skin application were sliced and the different layers (stratum corneum, epidermis, dermis, hypodermis) of each full thickness skin samples were separated, as previously reported [24], and homogenized to determine the amount of minoxidil.

### 2.16. In Vivo Minoxidil Determination at Hair Root Level

The amount of minoxidil penetrated into the hair follicles was determined in vivo. The experiment was carried out by applying on the back of rats 200 µL of the different minoxidil-loaded nanocarrier formulations (active compound concentration 0.5% *w*/*v*). The hydro-alcoholic formulation of the active compound was the control. The tape stripping methods was carried out by using a Tesa^®^ AG adhesive tape (Beiersdorf, Hamburg, Germany), for each site pre-treated for different times. A specific roller pressed the outlined sites, which naturally present wrinkles and furrows, to bring the tape strip on the entire flat skin surface area [28]. The same procedure was carried out in 3 animals for each group. After 1 h, 24 h, and 48 h of treatment, the formulation was gently removed and the tape was applied to sample the piliferous bulbs, which remained attached on it. The extraction of minoxidil from the tapes was carried out by using a mixture of ethanol:water (1:1 *v*/*v*) and hence stirring for 1 h at 190 rpm, as previously reported [29]. The removed active compound was determined by HPLC.

### 2.17. Hair Re-Growth Efficacy Test on Rats

The hair re-growth efficacy test was carried out on male Wistar rats (220 ± 10 g). Before starting the experiments, rats (*n* = 9) were shaved by electric shaver and they were distributed in three groups as follow: (i) untreated group considered as a control; (ii) group treated with a minoxidil hydro-alcoholic formulation (5% *w*/*v*); (iii) group treated with minoxidil-loaded LCNs (0.5% *w*/*v*). The animals were treated once a day for one month. At the end of the experiment, the hair length was measured in mm [30].

### 2.18. Anti-Hair Loss and Hair Re-Growth Efficacy Test by An Alopecia Animal Model

The efficacy of minoxidil-loaded LCNs (0.5% *w*/*v* in active compound) was also assayed on an alopecia animal thus enforcing the experimental evidence on the biological effect at the level of the pilosebaceous follicle (hair bulb). Male Wistar rats (*n* = 9) were distributed in three groups as previously described (see Section 2.17). Alopecia was induced on rats administering a cytostatic agent (0.2 mg/kg), doxorubicin [31]. Animals were treated once a day for 21 days, when a difference in the anagen phase of hair cycle was observed. Four days after stopping the treatment with doxorubicin, the experiments were carried out and samples were topically applied every day for 28 days thus evaluating the efficacy of formulation in the anti-hair loss and hair re-growth. Results were compared to those obtained following the treatment with a hydro-alcoholic minoxidil formulation at a concentration of 5% *w*/*v*.

### 2.19. In Vivo Toxicity Studies on Human Volunteers

The tolerability of empty LCNs was evaluated on human volunteers by using the reflectance spectrophotometer SP60 (X-Rite Incorporated, Grandville, MI, USA), which detects any changes in skin colour, regarding to the variations of the two main physiological chromophores present in human skin, i.e., melanin and hemoglobin [23]. The resulting data were expressed as the variation between erythema index values after and before the treatment (∆EI).

Twelve healthy human volunteers were enrolled in the experimental protocol and they gave their written informed consent, after receiving full details about the procedures and the study aim. Before the experiments, subjects rested for 30 min at room conditions (22 ± 2 °C; 40–50% relative humidity). The experimental protocol for the in vivo tolerability studies was as follows. Five sites were defined on the ventral surface of each forearm using a circular template (1 cm^2^). Erythema index (EI) baseline values were recorded before the application of samples at each site. Subsequently, 1 mL of formulation was applied using Hill Top chambers (Hill Top Re-search, Inc. Cincinnati, Miamiville, OH, USA) on each defined site. One site was treated only with saline solution (0.9% *w*/*v*) and used as a control. Before analysing the results, the chambers were gently removed and the skin was washed to eliminate any traces of formulation on the skin surface, so skin was allowed to dry 15 min. The induced erythema was monitored at 6, 12, 24, and 48 h, according to the following equation:

(6) $$EI=100[\log\frac{1}{R560}+1.5(\log\frac{1}{R540}+\log\frac{1}{R580})-2(\log\frac{1}{R510}+\log\frac{1}{R610})]$$

where *EI* is the erythema index, *1*/*R* represents the inverse reflectance at specific wavelength. Particularly, 560, 540, and 580 are the absorption peaks of hemoglobin, while 510 and 610 are the absorption peaks of melanin.

### 2.20. Ethics Statement

Studies involving human subjects were performed in accordance with the Declaration of Helsinki guidelines, and the protocols were approved by the Research Ethics Committee of the University “Magna Græcia” of Catanzaro (Ethics approval numbers: 390/2019; 392/2019).

### 2.21. Statistical Analysis

The statistical significance was evaluated by one-way ANOVA test, followed by Bonferroni post-test. The minimal level of significance in all experiments was set to *p* ≤ 0.05.

## 3. Results and Discussion

### 3.1. Physico-Chemical Characterization of Samples

The LCNs are hybrid nanocarriers made up of a block copolymer (polyethylene oxide-polypropylene oxide-polyethylene oxide) and phospholipid. The choice of the anionic phospholipid DMPG could influence the targeting characteristic of the final nanocarrier, since it gives an anionic charge, which can allow a better interaction and permeation through the skin [32,33]. Practically, LCNs belongs to Winsor IV microemulsion phase, and hence they are characterized by a great stability.

The confocal micrograph of the fluorescent labeled LCNs showed a particular tridimensional shape (Supplementary Figure S1), which is characterized by a highly organized biconcave structure, very similar to the red blood cells.

The physicochemical characterization is fundamental for the design and development of a new carrier because it greatly influences the biopharmaceutical properties of the delivered compounds. Especially for a topical administration, features such as mean size, polydispersity index and zeta potential could be decisive for the permeation through the skin [21,34]. For this reason, these features were investigated by using Zetasizer Nano ZS. Fluorescein and red oil, having different physic-chemical characteristics, hydrophilic and lipophilic respectively, were entrapped within LCNs as models of active compounds, enabling the evaluation of the carrier capacity and application potentialities of LCNs. The active compound minoxidil was also entrapped, giving the possibility to evaluate the application in the clinical practice.

Dynamic light scattering analysis (Table 1) showed that the various LCN formulations were characterized by mean sizes ranging from 80 to 100 nm, a narrow size distribution as evidenced by the low polydispersity index values and an LCN surface negatively charged. Altogether these features made LCNs suitable candidates for the topical skin administration and drug delivery. In particular, the encapsulation of hydrophobic and hydrophilic compounds, i.e., red oil and fluorescein, elicited a slight increase of the LCN mean size. The size increase seems to be related to two factors: (i) the molar mass of the encapsulated probes and (ii) their physicochemical features. Namely, the hydrophilic compound fluorescein seemed to be localized at the level of the outer layer of the LCNs, thus contributing with its negatively charged moieties to a slight increase of the LCN hydrodynamic radius as also evidenced by the zeta potential findings, i.e., a more negative zeta potential value. Meanwhile, the greatly lipophilic nature of oil red make it localize at the level of the LCN inner core, which is mainly hydrophobic, thus influencing the tight interaction between the hydrophobic part of the block-copolymer and the acyl chains of the phospholipid DMPG and hence leading to a greater disorganization of the packing order of the LCN core and as a consequence to a significant increase of the LCN mean size. In any case, as evidenced by the polydispersity index values, the size distribution of LCNs were not influenced by the presence of the three entrapped compounds. In contrast with fluorescein, oil red and minoxidil did not influenced the LCN zeta potential values due to their hydrophobic feature that make them confined in the inner part of the LCNs, thus having no contact with the nanocarrier surface.

Another important parameter to be evaluated to estimate the carrier capacity of LCNs is their entrapment efficacy regarding to the three different investigated compounds. As shown in Table 1, LCNs had great entrapment efficacy values with regard to all the three investigated compounds that, independently of their different physicochemical features, showed similar encapsulation efficiency values, thus evidencing that the LCN entrapment process was poorly influenced by the physicochemical nature of the compound and hence that LCN can be proposed as a carrier for both hydrophobic and hydrophilic compounds. This result, particularly in the case of fluorescein, was probably due to the presence of non-ionic surfactants in the LCN structure, which attenuate the repulsion forces between the negative charged DMPG and any negative charge of compounds, thus enhancing their encapsulation.

**Figure 1:**
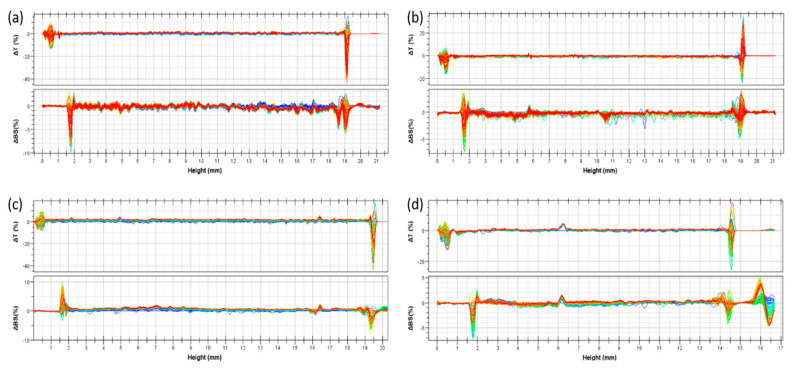
Stability profiles of LCNs by using Turbiscan Lab^®^ Expert. The various panels show the profiles of delta transmission (∆T) and delta back scattering (∆BS) as a function of time (0–3 h) and sample height (mm) at 24 ± 1 °C of the various investigated LCN formulations: (**a**) empty LCNs, (**b**) minoxidil-loaded LCNs at 0.5% *w*/*v* (as active compound), (**c**) fluorescein-loaded LCNs at 0.1% *w*/*v* and (**d**) red oil-loaded LCNs at 0.1% *w*/*v*.

The zeta potential of the various LCN formulations is also an index of their colloidal stability. In fact, the negative surface charge allowed a strong repulsion between the various nanocarriers, thus hampering any aggregation phenomenon. To have a further evidence of the LCN colloidal stability and to evaluate the storage stability, Turbiscan Lab^®^ analysis (Figure 1) was carried out by measuring the transmission and backscattering profiles as a function of time (0–3 h) and sample height (mm). This method allows to detect any kind of segregation phenomenon [35]. The obtained Turbiscan Lab^®^ data were consistent with zeta potential findings, thus showing that both empty and compound loaded LCNs were stable. Figure 1 shows the LCNs (liquid crystal nanocarriers) stability profiles. Regarding to the empty LCNs (panel a), minoxidil-loaded LCNs 0.5% *w*/*v* (panel b) and probe-loaded LCNs as 200 µL of a solution 0.1% *w*/*v* (panel c and d), the variation of backscattering profile was within values of ±5%, for the entire height of the samples, thus indicating that no migration phenomena occurs [23]. In fact, only a variation of backscattering profiles greater than 10%, as positive or negative values, can be considered as evidence of the presence of segregation phenomena which significantly alter the colloidal stability of a nanocarrier formulation [36].

### 3.2. In Vitro Bioassay on Human Cells

**Figure 2:**
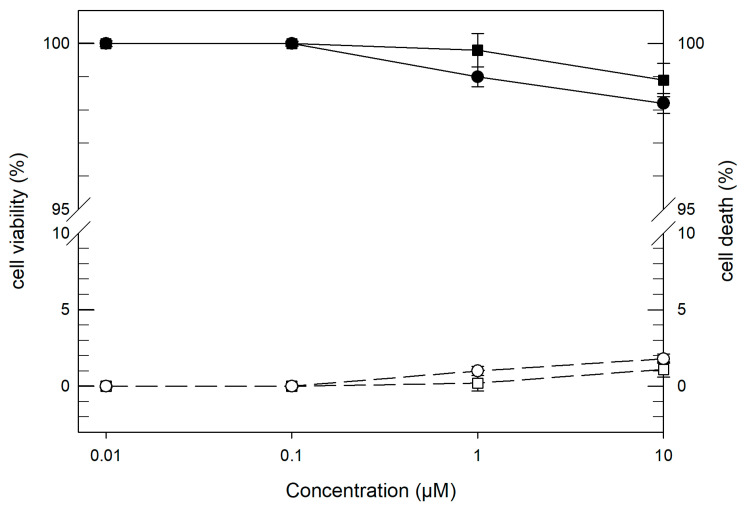
Cytotoxic effect of empty LCNs on human keratinocytes (NCTC2544) cells as a function of increasing lipid concentration after 72 h of incubation. LCNs cytotoxic effect is expressed both as percentage of cell viability (filled symbols and solid line, •) by MTT test and percentage of cell death (hollow symbols and dashed line, ◦) by trypan blue dye exclusion assay. For each experiment, untreated cells (■, □) were considered as control. Results represents the average of six different experiments ± standard deviation. If error bars are not visible, they are within the symbols.

The safety of LCNs was assayed in vitro in terms of cytotoxic effects on NCTC2544 keratinocyte cells (Figure 2). This cell line was chosen as a model for undifferentiated keratinocytes of the basal epidermal layer assuming a selective delivery to hair follicle, an epidermal appendage sunk into the dermis.

The toxicity was evaluated as a function of both LCN lipid concentration and nanocarrier incubation time, to determine the dose-dependent and exposition time-dependent effects. Mortality cell test showed the good safety of the carriers. That is to say, LCNs elicited a cell mortality of 1% at a concentration of 1 µM for all the incubation times (24, 48, 72 h) and a mortality cell values of 2% at a greater concentration (10 µM). These data were confirmed by the MTT cell viability test. In both tests, the untreated cells were used as control.

### 3.3. Percutaneous Permeation through SCE Membranes and Release Profiles

The capability of topical drug delivery systems to control pharmacokinetic parameters of the encapsulated drug has been well-described by many studies [12,37,38,39]. Therefore, the evaluation of percutaneous permeation and release profiles of active compounds from a new colloidal system is fundamental to define its efficacy. The Franz diffusion cells apparatus was used to assess both permeation and release profiles through SCE-membranes and synthetic membranes, respectively. A solution (200 µL, 0.1% *w*/*v*) of fluorescein (hydrophilic probe) or red oil O (hydrophobic probe) were encapsulated in LCNs and their release and percutaneous permeation profiles were compared to those obtained from two well-recognized colloidal delivery systems widely used as topical carriers for skin delivery [12,38,39,40], i.e., transfersomes^®^ and SLNs, loaded with the same amounts of the two probes.

**Figure 3:**
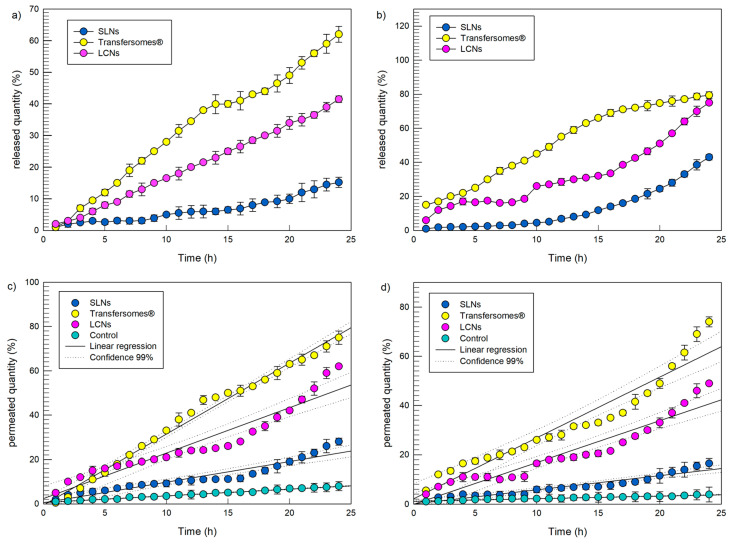
Release profiles (panel (**a**,**b**)) and percutaneous permeations (panels (**c**,**d**)) through SCE-membranes of the hydrophilic probe fluorescein (panels (**a**,**c**)) and the hydrophobic probe red oil O (panels (**b**,**d**)) by SLN, transfersomes^®^ and LCNs. Six different experiments were carried out and the results are expressed as the mean value ± standard deviation. If not visible, standard deviation bars are within the symbol.

The release (panels a and b) and percutaneous permeation (panels c and d) profiles of the investigated nanocarrier over 24 h are reported in Figure 3. The release data of both probes from the investigated nanocarriers showed the following decreasing release rate: transfersomes^®^ > LCNs > SLNs. Similar to the entrapment efficacy findings, the probe release rate was dependent on the nanocarriers rather than on the physiochemical features of the entrapped compounds. Interestingly, in the case of the hydrophobic red oil O (Figure 3b), the overall amount released over 24 h from LCNs was very close that released from transfersomes^®^.

The data of percutaneous permeation through SCE-membranes showed the same decreasing permeation order observed for the release experiments through artificial membranes.

The integrity of SCE-membranes was investigated before using membrane sheets and following the percutaneous permeation studies, using TEER measures. Any statistically significant variation of TEER values was recorded, thus demonstrating that skin sheets kept their integrity. In detail, values of TEER over 2 kΩ were recorded, that were consistent with previous studies [41].

Following the administration of samples, LCNs showed a significantly greater (*p* < 0.05) percutaneous permeation than SLNs, probably due to a preferential localization of the SLNs in the upper skin layers. Transfersomes^®^ showed the greatest percutaneous permeation thus confirming previous observations [42,43]. In fact, thanks to their characteristic deformability, transfersomes^®^ are able to improve the permeation of the delivered compounds through the human stratum corneum enabling them to reach the deeper skin layers, even if the exact mechanism by which they move through the skin layers remain to be elucidated [44,45].

Regarding to the delivered compound, LCNs provided suitable level of compound permeation through skin both in the case of the hydrophilic (~62% of the applied dose) and the hydrophobic (~50% of the applied dose) probe over 24 h, that is a fundamental requirement for topical application.

Fluxes of the two probes were also investigated to evaluate the quantitative percutaneous permeation through membrane sheets. In the case of red oil O the fluxes were 5.68 × 10^−3^ ± 3.03 × 10^−4^ μg/cm^2^ h^−1^, 3.05 × 10^−2^ ± 2.03 × 10^−3^ μg/cm^2^ h^−1^, 9.04 × 10^−2^ ± 6.11 × 10^−3^ μg/cm^2^ h^−1^, 1.32 × 10^−1^ ± 8.19 × 10^−3^ μg/cm^2^ h^−1^ for the probe solution, SLNs, LCNs, and Transfersomes^®^, respectively. Also, in the case of fluorescein, the analysis of the fluxes confirmed a greater percutaneous permeation in the case of the encapsulation of the hydrophilic probe in Transfersomes^®^. In particular, fluxes were 1.61 × 10^−2^ ± 2.94 × 10^−4^ μg/cm^2^ h^−1^, 4.97 × 10^−2^ ± 3.51 × 10^−3^ μg/cm^2^ h^−1^, 1.09 × 10^−1^ ± 7.58 × 10^−3^ μg/cm^2^ h^−1^, 1.71 × 10^−1^ ± 3.66 × 10^−3^ μg/cm^2^ h^−1^ for the probe solution, SLNs, LCNs, and Transfersomes^®^, respectively.

To evaluate the preferential pathway followed by the different investigated nanocarriers, in vivo studies were performed.

### 3.4. In Vivo Experiments in Rats

The permeation through the skin can occur by different pathways: (i) across epithelial cellular membrane (transcellular), (ii) between the cells (paracellular), (iii) via sweat glands or pilosebaceous follicles/hair follicles (appendageal route) [46]. For this reason, the main permeation pathway followed by the studied nanocarriers (transfersomes^®^, SLNs, and LCNs) was investigated. The preferential use of a specific permeation pathway, especially in the case of the appendageal route, can be of strategic relevance in the clinical practice to achieve a site-specific delivery of active compounds. Interestingly, the site-specific delivery of active compounds to pilosebaceous follicles can allow more efficient treatment of a number of diseases and/or disfunctions, thus reducing the administered dose and hence the possible appearance of side effects.

**Figure 4:**
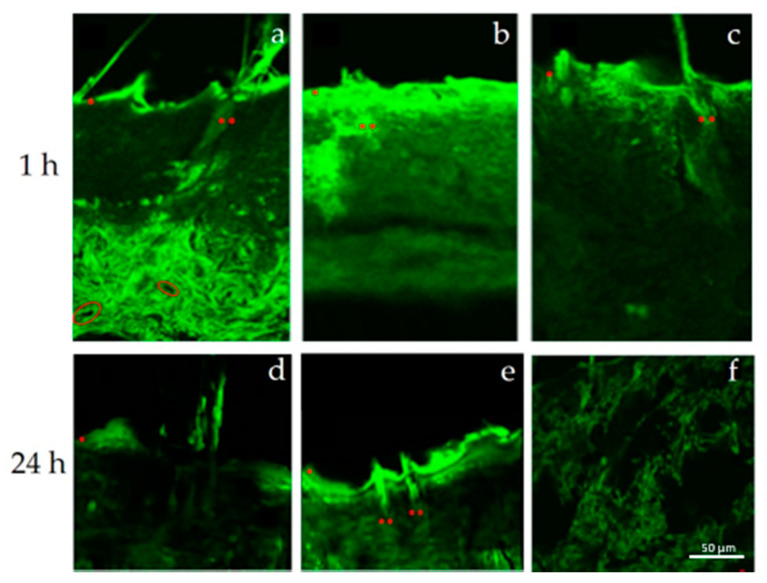
Micrographs of skin sections after 1 h and 24 h from the treatment with the various fluorescein-loaded nanocarriers. Keys: (**a**) transfersomes^®^, (**b**) SLNs, (**f**) SLNs, superficial view (**c**) LCNs, (**d**) up and (**e**) down of the skin section treated with transfersomes^®^. Some skin elements are highlighted with the following symbols: * stratum corneum (SC), ** pilosebaceous follicle, circle: blood vessels.

Figure 4 shows the permeation through the skin in serial sagittal microsection 1 h after the application of fluorescein-loaded various nanocarriers. The micrographs show a preferential localization of fluorescein loaded LCNs toward the pilosebaceous follicle and a wide dispersion in the skin layers of transfersomes^®^ and SLNs. In particular, transfersomes^®^ (Figure 4a) permeate the stratum corneum up to the deeper skin layers, probably due to their intrinsic ultra-flexibility [47]. In fact, when applied in non-occlusive condition they are able to easily pass through the stratum corneum and to deliver substances, as a function of the hydration gradient of the skin without damaging it [48]. The wide fluorescent probe dispersion in the skin layers when delivered by transfersomes^®^ and SLNs was still detectable after 24 h (Figure 4). The wide cutaneous dispersion levels suggest that these two nanocarriers are not suitable to guarantee a specific effect in the pilosebaceous unit. Moreover, the non-specific long stay of solid lipid nanoparticles within the skin layers could also lead to irritation phenomena.

**Figure 5:**
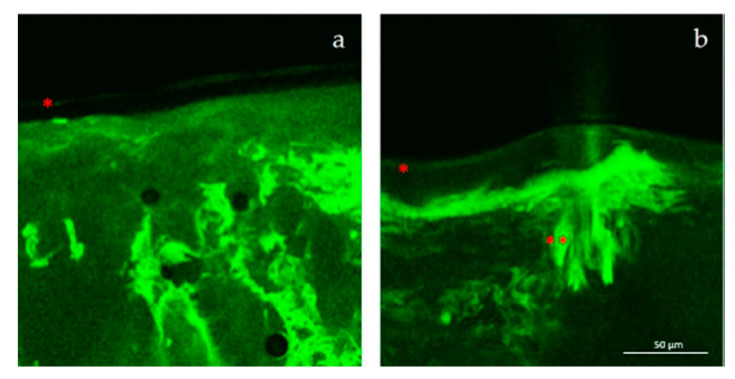
Confocal micrographs of skin sections after 24 h from the treatment with fluorescein-loaded LCNs, highlighted the different accumulation of the fluorescent probe between the hair bulb (**a**,**b**) or the deeper skin layers (**b**) * stratum corneum (SC), ** pilosebaceous follicle.

Contrariwise, LCNs showed a specific pilosebaceous follicular targeting ability, also at 24 h after treatment. The massive presence of the fluorescent probe in the hair bulb (Figure 5a,b) was accompanied by the absence of the probe in the deeper skin layers and in the blood vessels (Figure 5b). This finding documented that LCNs can be considered as suitable systems for the follicular active ingredient delivery, with the potential to minimize the loss of loaded active compounds and systemic side effects.

### 3.5. In Vivo Experiments on Rats Using Minoxidil-Loaded Nanocarriers

**Figure 6:**
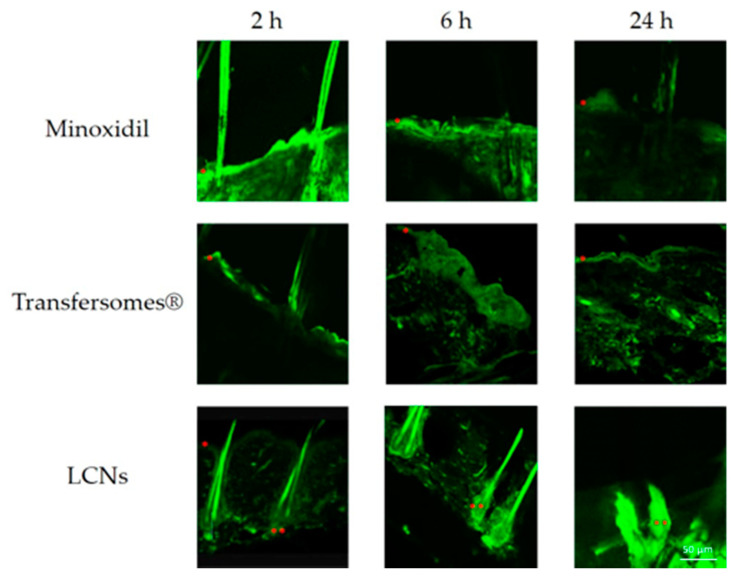
In vivo pilosebaceous delivery of minoxidil following the skin treatment with a hydro-alcoholic, transfersomes^®^ or LCNs formulations as a function time (2, 6, 24 h from the treatment). * stratum corneum (SC), ** pilosebaceous follicle.

Minoxidil, an active compound that is able to modulate the hair loss and regrowth, was encapsulated (0.5% *w*/*v*) in transfersomes^®^ and LCNs to compare their capability to reach the deeper skin layers and deliver actives into the pilosebaceous unit. The skin distribution of minoxidil can be easily detected owing to its fluorescence property. Micrographs of the skin of untreated rats (Figure S2) showed the very low autofluorescent phenomena occurring when the nanosystems have not been administered. As can be seen in Figure 6, the hydro-alcoholic formulation of minoxidil was not able to selectively reach its target, in fact it was widely distributed in the different skin layers and it was rapidly metabolized, in fact a very low fluorescence has been detected after 6 h from the treatment. In contrast, minoxidil-loaded LCNs had a greater targeting capacity to the pilosebaceous unit as well as a longer in situ lifetime (until 24 h). Meanwhile, minoxidil-loaded transfersomes^®^ were characterized by a random distribution in the skin and an increase of a non-selective percutaneous permeation.

These experiments shed light on the ability of LCNs to ensure a hair bulb targeting of minoxidil, thus suggesting an improvement of the biological effectiveness as well as a reduction of the side effects related to the topical administration which, in the case of minoxidil, can be very serious [20,49].

### 3.6. Quantification of Minoxidil at Hair Root Level

The tape stripping test was carried out on rats to evaluate the amount of minoxidil that actually penetrated into the pilosebaceous unit. As shown in Table 2, all time points (1 h, 24 h, 48 h) showed a certain follicular bioavailability of minoxidil-loaded LCNs (91–74% of the applied dose) than minoxidil-loaded other nanocarriers or its hydro-alcoholic formulation. In particular, after 1 h from the skin topical treatment, the amount of minoxidil delivered to the level of the pilosebaceous follicle by LCNs was seven and two times greater (*p* < 0.001) than those by the hydro-alcoholic formulation and transfersomes^®^ or SLN, respectively. Even if transfersomes^®^ and SLNs also increased the amount of minoxidil at hair root level with respect to the hydro-alcoholic formulation (*p* < 0.05), only LCNs were able to ensure the longest in situ retention time of the delivered active compound. Namely, a significant (*p* < 0.001) minoxidil amount (74% of the administered dose) was still observed at the level of the hair follicle after 48 h from the topical administration, while a very low amount was observed for all other formulations.

After 1 h of treatment, the percentage increase of active delivery efficiency obtained for minoxidil-loaded carriers reached values of ~600% in the case of colloidal liquid crystals, compared to the free active (see Supplementary Figure S3).

**Figure 7:**
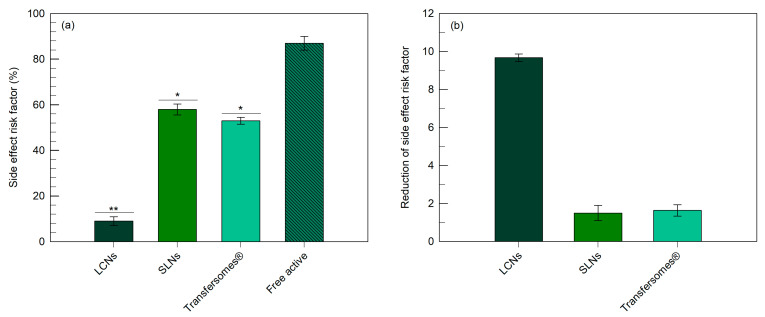
Evaluation of the side effect risk factor (panel (**a**)) (measured as the percentage of the minoxidil topically administered dose that did not reach the hair bulb) and the reduction of side effect risk factor (panel (**b**)) (measured as the ratio between the nanocarrier risk factor and that of the hydro-alcoholic formulation). The data obtained for minoxidil-loaded LCNs were statistically significant with respect to those obtained for SLNs, transfersomes^®^ and the hydro-alcoholic formulation (* *p* < 0.05; ** *p* < 0.001).

The side effects risk factor was also calculated as the percentage of active compound that did not reach the physiological target (Figure 7a). This value was of ~9% for LCNs, a significantly lower (*p* < 0.001) than for the hydro-alcoholic formulation. The reduction of side effects risk factor (Figure 7b) was calculated as a ratio between the side effects risk factor of the various nanocarriers and that of the hydro-alcoholic formulation. In particular, the reduction of side effects risk factor was significantly (*p* < 0.001) lower (9.7 times) for LCNs than those for SLNs and transfersomes^®^. No significant difference was observed for the reduction of side effects risk factor between SLNs and transfersomes^®^. These data show the ability of LCNs to potentially minimize the systemic side effects of the delivered active compound.

### 3.7. In Vivo Minoxidil Skin Layers Distribution

To further investigate the amount of minoxidil localized at the various skin layers, studies of percutaneous permeation were performed on rats. In detail, full thickness skin sections were collected after 24 h from the topical treatment to quantify the minoxidil skin layers distribution, using HPLC method. Table 3 shows the concentrations of the active compound in epidermis, dermis and hypodermis following the topical treatment with the different formulations.

The overall cutaneous concentration of the active compound following the topical skin treatment with minoxidil-loaded LCNs was double (~84% of the administered dosage) with respect to that obtained with the hydro-alcoholic solution (~42% of the administered dosage). In particular, the following overall cutaneous skin accumulation decreasing order was observed: LCNs > transfersomes^®^ > SLNs > hydro-alcoholic formulation. In addition to the overall minoxidil skin accumulation that was different as a function of the topical formulation, also the active compound distribution varied between the various skin layers. Specifically, minoxidil-loaded LCNs were predominantly recovered in the dermis, where the pilosebaceous units are mostly present [50], thus further confirming the previous findings (see Section 3.6). Transfersomes^®^ provided a homogeneous minoxidil distribution between the skin layers with a significant amount at the level of hypodermis, thus evidencing the concrete possibility to reach the bloodstream and hence to develop systemic side effects. A significant amount of minoxidil in the hypodermis was also observed following the treatment with the hydro-alcoholic formulation. A minoxidil localization at the level of the upper skin layers was observed in the case of SLN treatment, i.e., 77% of the overall administered dose was recovered in the epidermis. Therefore, according to these findings, the LCNs seems to be the most suitable delivery device for the treatment of follicular diseases due to their selective targeting towards the pilosebaceous follicle. For this reason, the minoxidil-loaded LCNs were chosen for the in vivo efficacy test in comparison with the hydro-alcoholic formulation.

The overall concentration of minoxidil was also measured after 24 h from application (Supplementary Figure S4).

### 3.8. In Vivo Efficacy Test

**Figure 8:**
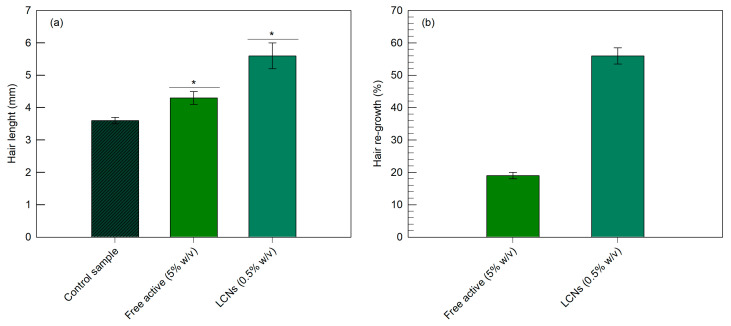
Evaluation of the re-growth effectiveness in rats following the treatment with the minoxidil-loaded LCNs or its hydro-alcoholic formulation. The re-growth efficacy was expressed both as measure of the hair length (panel (**a**)) and percentage of the hair length increase (panel (**b**)) in comparison with the control. The data obtained for minoxidil-loaded LCNs were statistically significant with respect to the control (* *p* < 0.001).

In vivo efficacy tests were carried out on rats by measuring the length of the hair (in mm) following the topical treatment with the minoxidil-loaded LCNs or the hydro-alcoholic formulation. As shown in Figure 8a, untreated rats had a mean re-growth of 3.6 mm after one month. Animals treated with the hydro-alcoholic minoxidil formulation (5% *w*/*v*) showed a mean re-growth of 4.3 mm, while those treated with minoxidil-loaded LCNs (0.5% *w*/*v*) had a significantly (*p* < 0.001) longer mean re-growth (5.6 mm). The improvement of the re-growth effect, expressed as the percentage of hair length increase (Figure 8b) with respect to the control (untreated animals), was 56% and 19% following the treatment with minoxidil-loaded LCNs and its hydro-alcoholic formulation, respectively. It should be taken into due consideration that the hydro-alcoholic formulation has an active compound concentration ten-times greater than LCNs, thus evidencing the improved biological efficacy of minoxidil-loaded LCNs as well as a significant reduction of the occurrence of systemic side effects, which are directly related to the administered dose [51].

**Figure 9:**
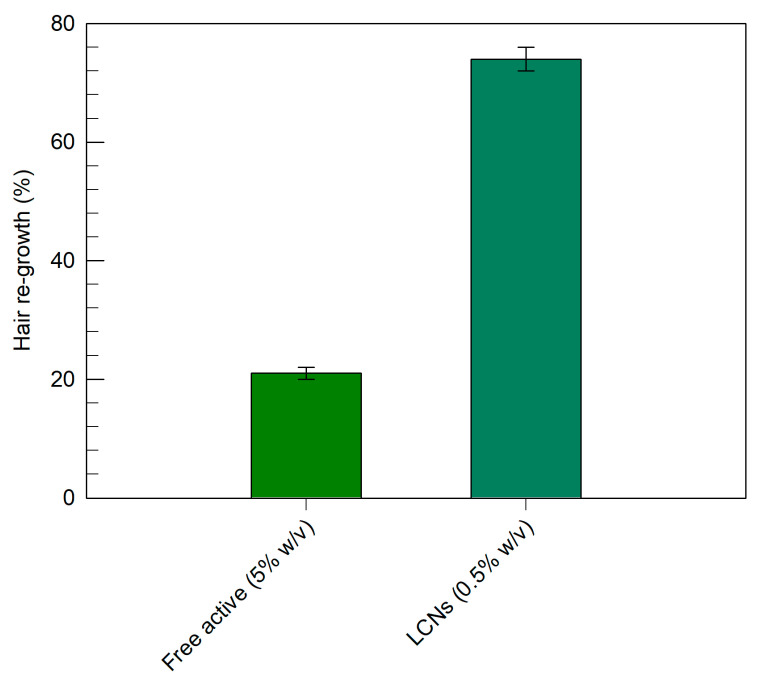
The re-growth efficacy expressed as percentage of the hair length increase in comparison with the control following the treatment with the minoxidil-loaded LCNs or its hydro-alcoholic formulation. The data obtained for minoxidil-loaded LCNs were statistically significant with respect to the hydro-alcoholic formulation (*p* < 0.001).

To reinforce the evidence of the biological effectiveness of minoxidil-loaded LCNs, in vivo experiments on an alopecia animal model were also carried out. These findings were in agreement with those reported in Figure 8, confirming the most suitable biological activity of minoxidil-loaded LCNs. Of note, in the animal model of alopecia (Figure 9), the minoxidil-loaded LCNs showed even greater biological activity than that observed in the above reported experiments, resulting in 74% of hair regrowth, when compared to the controls.

### 3.9. LCN Safety in Human Volunteers

The in vivo tolerability regarding LCNs was tested by monitoring the erythema index variation (∆EI) before and after treatment. The experiments were carried out on 12 healthy human volunteers until 48 h and results were reported as the mean values ± standard deviation.

**Figure 10:**
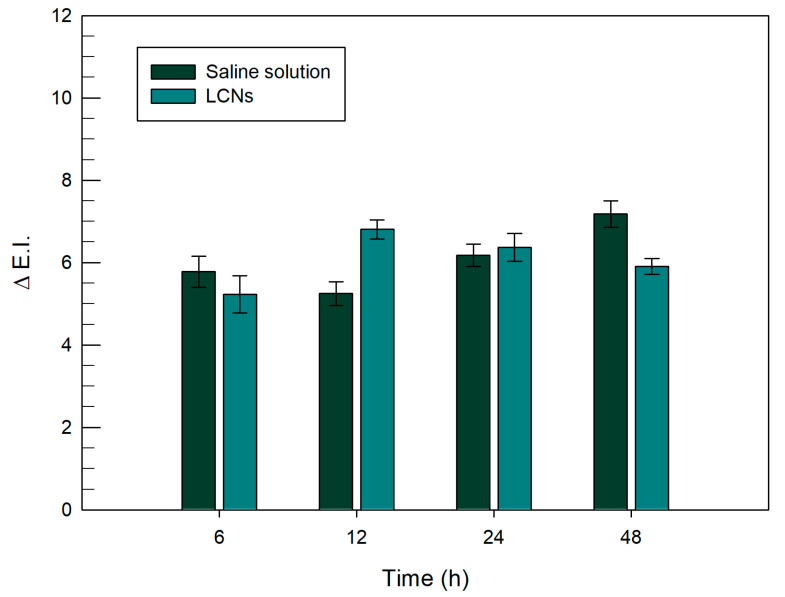
In vivo skin tolerability studies performed on human healthy volunteers. The unloaded LCNs were applied on the ventral surface of each forearm of volunteers and the variation of erythematous index was monitored as a function of the exposition time, i.e., 6, 12, 24 and 48 h. Data are expressed as the mean value ± standard deviation.

As shown in Figure 10, the obtained ∆EI values did not vary significantly for all timepoints, compared to the control (saline solution). A variation below 1.5 was recorded for all exposition times and this was probably related to the composition of the LCNs, which were made up of biocompatible ingredients. In fact, the main component, glycerol monooleate, was classified as GRAS (generally recognized as safe) and included in the FDA Inactive Ingredients Guide [52], reducing the probability of toxic events generally due to aggressive excipients such as surfactant mixtures.

Moreover, there was no macroscopically visible erythema on the sites treated with empty colloidal liquid crystals until 48 h from application. The in vivo results of studies performed on healthy human volunteers confirmed the in vitro studies on NCTC2544 (see Section 3.2) and documented the suitable level of tolerability and safety of this nanocarrier.

## 4. Conclusions

The LCNs investigated in this study are suitable carriers for a selective delivery of active compounds towards the pilosebaceous follicle. These supramolecular carriers are stable, safe, able to effectively contain and release active compounds, and to successfully permeate the skin. The great advantage of LCNs emerging from this investigation, is their concrete possibility to be used in the clinical practice for the topical site-specific active compound delivery thanks to their pilosebaceous follicle targeting features. The pilosebaceous follicle targeting seems to be a peculiarity of the LCNs herein investigated with respect to other widely used topical delivery systems, i.e., transfersomes^®^ and SLNs, which provided an indiscriminate skin distribution of the delivered compounds, mostly in the lower and upper skin layers, respectively.

The pilosebaceous targeting was also evidenced in an animal model, that showed an improved biological effectiveness in terms of hair re-growth following the treatment with minoxidil-loaded LCNs. LCNs also showed the advantage of potentially reducing the active compound side effects due to two factors: (i) the low amount of active compound normally transferred into the circulatory system and (ii) the use of a one-tenth dose with respect to the minoxidil hydro-alcoholic formulation available on the market. In fact, our results showed that the LCNs can be administered in lower dosages than those of conventional therapy using minoxidil lotion, also because of a longer pilosebaceous follicle retention time. These findings are very encouraging and suggest the use of LCNs for the treatment of different diseases affecting the pilosebaceous unit.

## Figures and Tables

**Table 1:** Physico-chemical features and entrapment efficiency of LCNs entrapping three different compounds (having different physicochemical characteristics) in comparison with empty LCNs ^a^.

LCN Sample	Concentration (% *w/v*)	Mean Size (nm)	Polydispersity Index	Zeta Potential (mV)	Entrapment Efficacy (%)
Empty	-	85 ± 1	0.20 ± 0.01	−55 ± 4	-
Fluorescein-LCNs	0.1	92 ± 1	0.18 ± 0.01	−68 ± 2	97 ± 2
Red oil O-LCNs	0.1	101 ± 1	0.19 ± 0.02	−59 ± 3	96 ± 2
Minoxidil-LCNs	0.5	82 ± 1	0.15 ± 0.00	−57 ± 3	98 ± 1

^a^ Each value is the mean of three experiments ± standard deviation.

**Table 2:** Amount of minoxidil at the level of the pilosebaceuos follicle following its topical administration as active compound-loaded nanocarriers or hydro-alcoholic formulation as a function of time. The minoxidil amount was expressed as recovered percentage with respect to the initial applied dose ^a^.

Stripping Test	LCNs	SLNs	Transfersomes^®^	Hydro-Alcoholic Formulation
After 1 h	91% ± 7	42% ± 9	47% ± 8	13% ± 8
After 24 h	86% ± 9	24% ± 7	31% ± 9	11% ± 7
After 48 h	74% ± 8	18% ± 7	13% ± 7	1% ± 0

^a^ Each result is the mean of three different experiments ± standard deviation. The data obtained for minoxidil-loaded LCNs were statistically significant (*p* < 0.001) with respect to those obtained for SLNs, transfersomes^®^ and hydro-alcoholic formulation. Minoxidil bioavailability is expressed as the percentage of the administered dose.

**Table 3:** Minoxidil amount at the different skin layers following its topical administration as active compound-loaded nanocarriers or hydro-alcoholic formulation. The minoxidil amount was expressed both as recovered percentage with respect to the initial applied dose ^a^.

Different Skin Layers	LCNs	SLNs	Transfersomes^®^	Hydro-Alcoholic Formulation
1–2 mm (epidermis)	19% ± 5	37% ± 6	21% ± 7	9% ± 4
4–5 mm (dermis)	58% ± 4	9% ± 5	18% ± 5	12% ± 4
10–11 mm (hypodermis)	7% ± 4	2% ± 1	15% ± 6	21% ± 6

^a^ Each results is the mean of three different experiments ± standard deviation. The data obtained for minoxidil-loaded LCNs were statistically significant (*p* < 0.001) with respect to those obtained for SLNs, transfersomes^®^ and hydro-alcoholic formulation. Minoxidil bioavailability is expressed as the percentage of the administered dose.

## Supplementary Materials

The following are available online at https://www.mdpi.com/1999-4923/12/11/1127/s1, Figure S1: Confocal micrograph of fluorescently labeled LCNs; Figure S2: CLSM micrograph of the skin of untreated rats. A: transmittance; B: FITC channel; Figure S3: Increase of active delivery efficiency, measured as percentage increase of active concentration at follicular site after 1 h of treatment (the free active measurement was used as reference). The data obtained for minoxidil-loaded liquid crystal nanocarriers were statistically significant with respect to that obtained from the free active; Figure S4: Permeation studies at different skin layers. Overall concentration of minoxidil measured after 24 h from application. Results were expressed as percentage of the initial dosage applied. The data obtained for minoxidil-loaded LCNs were statistically significant with respect to those obtained from the free active (* *p* < 0.05; ** *p* < 0.001).
