# Supplementary material for: Targeting of the Pilosebaceous Follicle by Liquid Crystal Nanocarriers: In Vitro and In Vivo Effects of the Entrapped Minoxidil
Source: Pharmaceutics. 2020 Nov 22;12(11):1127. doi: 10.3390/pharmaceutics12111127 (PMC7700400; doi:10.3390/pharmaceutics12111127)
Supplement: Supplementary file 1 [file pharmaceutics-12-01127-s001.pdf]

pharmaceutics

Supplementary Materials: Targeting of the Pilosebaceous Follicle by Liquid Crystal Nanocarriers: in Vitro and in Vivo Effects of the Entrapped Minoxidil

Massimo Fresta, Antonia Mancuso, Maria Chiara Cristiano, Konrad Urbanek, Felisa Cilurzo, Donato Cosco, Michelangelo Iannone and Donatella Paolino*

Figure S1 shows a confocal micrograph of the fluorescent labeled LCNs. The nanosystems were characterized by a highly organized biconcave structure, very similar to the red blood cells.

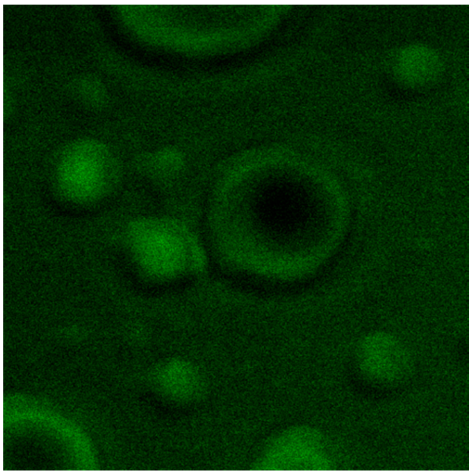

Figure S1. Confocal micrograph of fluorescently-labeled LCNs.

Figure S2 presents a micrograph of the skin of untreated rats in order to demostrate the very low autofluorescence that occurs when the nanocarriers have not been administered.

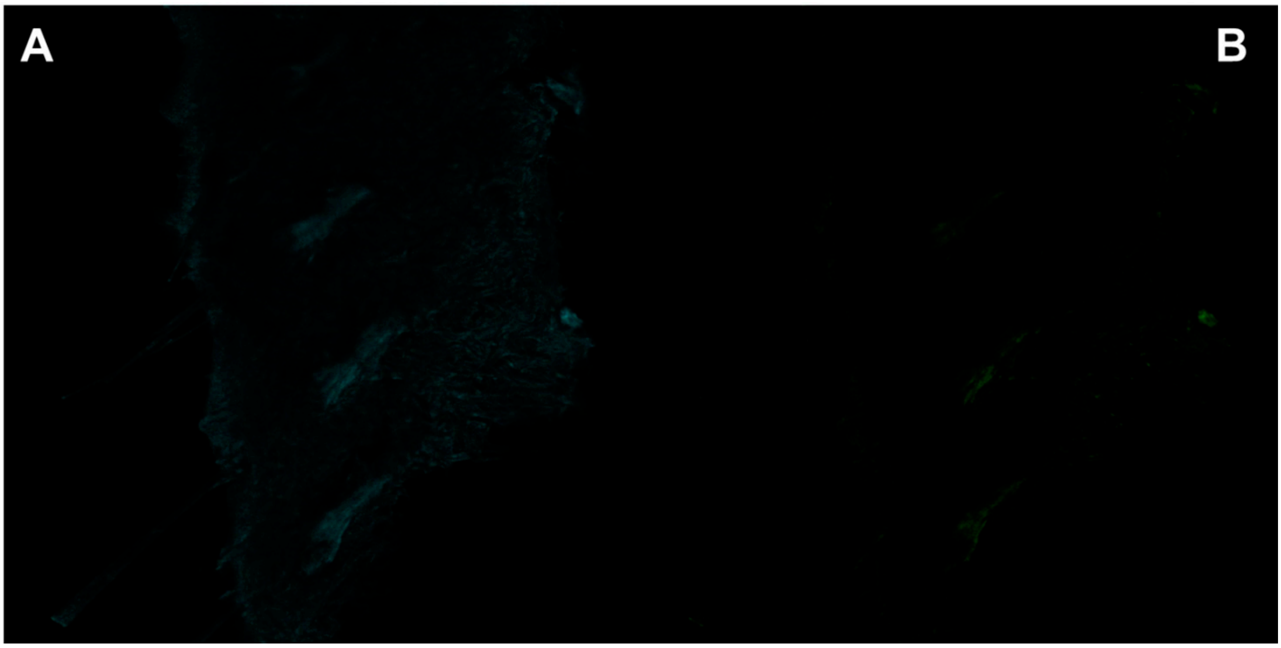

Figure S2. CLSM micrograph of the skin of untreated rats. **A**: transmittance; **B**: FITC channel.

Figure S3 reports the percentage increase of active delivery efficiency obtained for minoxidil-loaded nanocarriers after 1 h of treatment. As can be seen, minoxidil-loaded LCNs reached values of ~600%, compared to their free active counterparts. The percentage values recorded for minoxidil-loaded transfersomes® and SLNs were about half of those obtained from minoxidil-loaded LCNs. The obtained results highlight the better ability of LCNs to deliver active substances at the follicular site, in comparison to both the hydro-alcoholic solution of minoxidil and to the other active-loaded analyzed carriers, i.e., SLNs and transfersomes®.

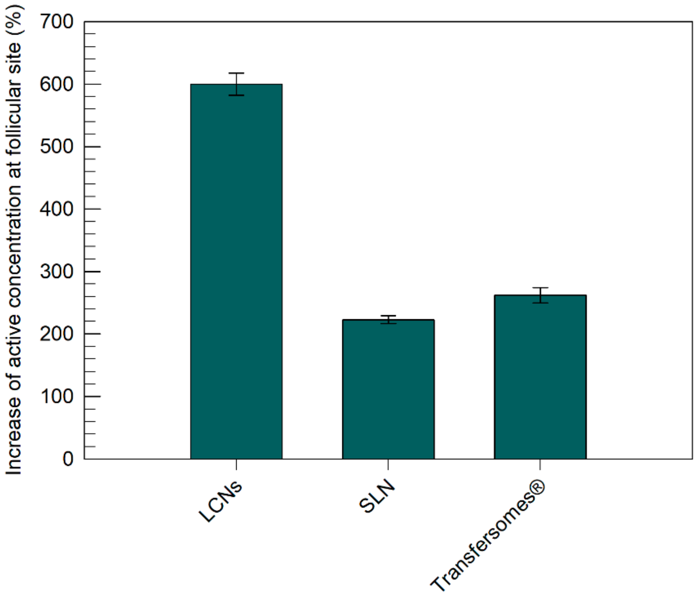

Figure S3. Increase of active delivery efficiency, measured as percentage increase of active concentration at follicular site after 1 h of treatment (the free active measurement was used as reference). The data obtained for minoxidil-loaded liquid crystal nanocarriers were statistically significant with respect to that obtained from the free active.

Figure S4 shows the overall concentration of minoxidil at different skin layers after 24 h of treatment. Minoxidil-loaded LCNs showed percentage values significantly greater with respect to those obtained from the free active and the other active-loaded analyzed carriers.

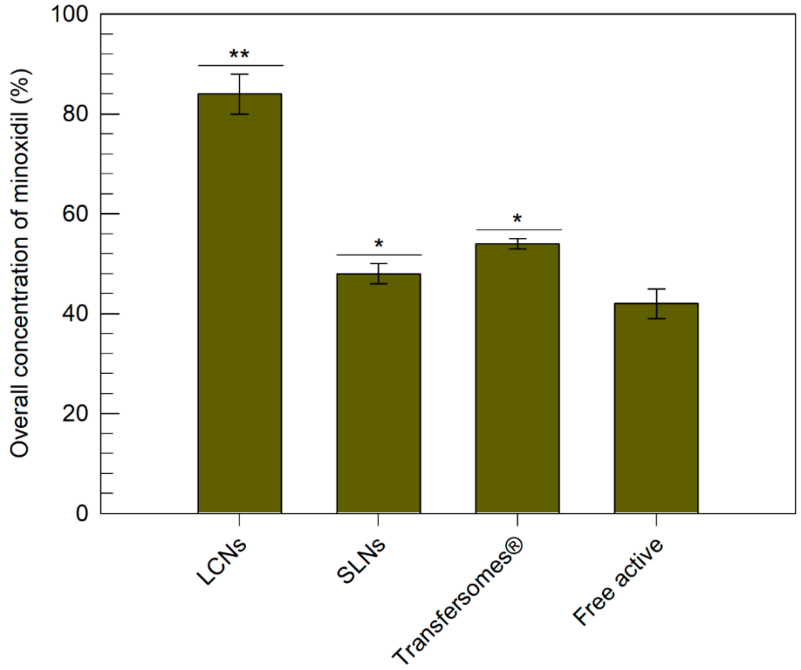

Figure S4. Permeation studies at different skin layers. Overall concentration of minoxidil measured after 24 h from application. Results were expressed as percentage of the initial dosage applied. The data obtained for minoxidil-loaded LCNs were statistically significant with respect to those obtained from the free active (* $p < 0.05$; ** $p < 0.001$).
